# A topology control algorithm for fusion networks based on link quality

**DOI:** 10.1016/j.mex.2023.102423

**Published:** 2023-10-10

**Authors:** Wei Lian Suo, Huan Shen Hao, Ma Long Yu

**Affiliations:** School of Information Engineering, Suqian University, SuQian, Jiangsu 223800, China

**Keywords:** A Network Topology Control Algorithm Based on the Fusion of Thiel Entropy and Potential Game Theory(PG-ORTC), Wireless sensor network, Theil entropy, Potential game, Topology control

## Abstract

To extend the network life, an ordinal potential game is introduced into network topology control, and a network topology control algorithm based on the Theil entropy measure is designed by improving the revenue function. The revenue function is based on the Theil entropy measure, whose factors are in the residual energy of the nodes and their neighbors. By using a primary payoff function that considers all initial network factors and a secondary one that addresses connectivity in case of reduced residual energy, a segmentation function can calculate the model's payoff. Simulation experiments show that compared to the existing game algorithms of 3DK-RNG, DEBA, and EFPC, the proposed algorithm can effectively eliminate redundant links, reduce node degree and network link length, balance node energy consumption, enhance network load, and extend the network life cycle.

• A Network Topology Control Algorithm Based on the Fusion of Thiel Entropy and Ordinal Potential Games.
• A network topology control method for extending the network lifecycle is successfully established.
• It proposes that the performance of the fusion topology control algorithm is significantly superior to other single algorithms in the paper.

## Graphical abstract

**Figure:**
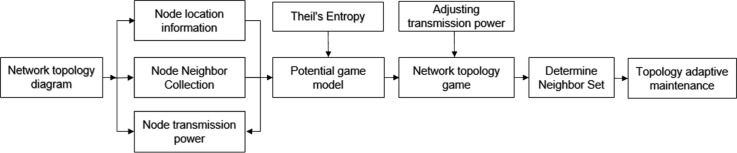

Specifications table

**Table:** 

Subject area:	Computer Science
More specific subject area:	Network Topology Control
Name of your method:	A Network Topology Control Algorithm Based on the Fusion of Thiel Entropy and Potential Game Theory(PG-ORTC)
Name and reference of the original method:	*N/A*
Resource availability:	*N/A*

**Method details**

## Introduction

Wireless sensor networks are self-organizing networks composed of a large number of battery-powered sensor nodes with limited energy [1], [2]. They are widely used for marine information collection and disaster detection. Topology control algorithms [3], [4] are researching primarily on technology in wireless sensor networks, which optimizes transmission power, eliminates redundant links, reduces network energy consumption, extends network life cycles, and improves service quality while maintaining network connectivity. Currently, many researchers [5], [6] have proposed related topology control algorithms and achieved exceptional results in wireless sensor networks. The current focus on energy consumption has led to neglect of network connectivity [7].

This paper also focuses on the performance study of many different network topological settings for mission-critical applications. Lavanya and Shanmugapriya [8] proposed the DIA algorithm that used game theory and selfish node behavior to design an efficient topology. The proposed algorithm optimizes node-transmitting power and improves network performance using distributed dynamic response. However, it was limited to terrestrial wireless sensor networks and failed to consider wireless factors. Kaswan et al. [9] proposed a particle swarm optimization-based clustering algorithm with a mobile sink for wireless sensor networks. Wang et al. [10] proposed the 3DK-RNG algorithm for 3D wireless sensor networks to ensure nodes are always k-connected and make the network robust. However, it neglected node degree and caused energy depletion in many nodes. Gu et al. [11] proposed the EFPC algorithm, using game theory to improve topology control and optimize the performance of wireless sensor networks with multiple wireless disturbances. To alleviate unnecessary energy wastage and improve network performance, Wang et al. [12] introduced a novel coverage control algorithm based on particle swarm optimization. However, the ‘hot spots’ problem was caused since nodes near the sink would consume more energy during forwarding. Wang et al. [13] proposed an improved particle swarm optimization combined with a mutation operator, which was introduced to search the parking positions with optimal coverage rate. Then the genetic algorithm was adopted to schedule the moving trajectory for multiple mobile sinks.

Although the above algorithms considered the uncertainties in wireless sensor networks and their impacts on algorithm performance, they did not consider energy consumption and transmission delay that affect the life cycles of algorithms. Nouir et al. [14] proposed to use of fuzzy logic to select free sensor nodes and CHs with four fuzzy parameters. These parameters are the energy level of the sink and sensor proximity to the sink in terms of free sensors selection, and the energy level of the sensor node and centrality of sensors in terms of CHs selection. Lee and Teng [15] proposed an improved low-energy adaptive clustering hierarchy protocol for mobile sensor networks to not only prolong the network lifetime but also reduce packet loss using a fuzzy inference system. However, this approach ignored the residual energy of nodes, resulting in the imbalance of network energy consumption. Liu and Qo [16] introduced the nearest neighbor graph into network topology control and designed the *EBTCA* algorithm, comprehensively considering both the remaining energy and the link length of nodes to enhance the robustness of the network, balance the network load, and extend the network life.

However, topology control algorithms sometimes overemphasize energy balance at the cost of connectivity, leading to low network robustness in wireless sensor networks. Priya and Mohanraj [17] proposed an energy-aware clustering and efficient cluster head selection (*EAC-ECHS*) to optimize the performances of wireless sensor networks in terms of network lifetime and enhance energy consumption. The algorithm took into account not only the residual energy of nodes but also the link quality of the network, thus balancing energy consumption and enhancing network robustness. Lee and Yang [18] proposed the DEBA topology control algorithm, intended to balance network energy consumption using a function. Though it reduced energy consumption in nodes, it negatively affected network connectivity and robustness. Wang et al. [19] introduced a multiple-strategies differential privacy framework on STF (MDPSTF) for HOHDST network traffic data analysis. MDPSTF had high universality on the various degrees of privacy protection demands and high recovery accuracy for the HOHDST network traffic data. Aiming at the above problems, the PG-ORTC was designed utilizing the ordinal potential game in wireless sensor networks. The paper introduces the Theil entropy measure to enhance the revenue function and improve the measurement of energy consumption of nodes. It also incorporates energy consumption, connectivity, transmission delays, and other factors to ensure a comprehensive consideration. The comprehensive benefit function is considered primary over the connectivity function, which is secondary, to minimize network energy consumption and prolong survival time. Simulation experiments indicate that our proposed algorithm outperforms 3DK-RNG, DEBA, and EFPC.

The paper is organized as follows. In Sec.Ⅱ, the model and related theory are reviewed. A network topology control algorithm based on Theil's entropy measure is designed by improving the revenue function is discussed in Sec. Ⅲ. Sec. Ⅳ is devoted to analyzing the system performance through simulation example and studying the proposed algorithm, which reduced the number of nodes’ deaths and the rate of a network fault. The proposed algorithm has also proved to have done a good performance in terms of the survival number of nodes, network traffic, and the prolongation of network lifetime. Conclusions are summarized in Sec. Ⅴ.

## Model and related theory

Based on the relevant principles of graph theory, the network topology can be represented as an undirected graph in this paper. Where denotes the set of *N* nodes deployed; $P=\left(p_{1},p_{2},\cdots ,p_{N}\right):p_{i}\in \left[p_{i}^{\min},p_{i}^{\max}\right]$ denotes the set of communication transmitting power of *N* nodes within their communication range; $p_{i}^{\min}$is the threshold value of transmitting power of node *i*, $p_{i}^{\max}$ is the maximum transmitting power of node *i*.$E=\{e_{i}^{j},i\in N,j\in N,i\neq j\}$, which denotes the set of links for communication between two random nodes i and node *j*. To enable experimental simulation analysis, we impose the following constraints on the three-dimensional wireless sensor network:

(1) The impact of boundary constraints on wireless sensor network monitoring can be neglected.
(2) The sensor node's transmission range is determined by *r*(*i*), also known as the communication radius, and can be adjusted according to its application. The node's transmission range forms a spherical boundary with *r*(*i*) as its radius.
(3) Assuming that each randomly deployed sensor node demonstrates rationality and selfishness.
(4) The lifetime of a wireless sensor network is determined by the time that takes the first node to become dead.
(5) The initialization process for each node (*i*) is identical, except for the node ID, which is unique in a wireless sensor network.
(6) A randomly selected node can determine the connectivity of a wireless sensor network.
(7) The three-dimensional wireless sensor network enables half-duplex signal transmission between node *i* and node *j*.

Measuring the sensor nodes is difficult due to the unpredictable wireless network environment. Therefore, a systematic evaluation of several factors is necessary to scrutinize network topology, as described below:

(1) Network connectivity: Network connectivity is a crucial requirement for a complete wireless sensor network topology. After multiple rounds of optimizing the sensor nodes, it is essential to ensure that the network is still connected. Otherwise, any further conducted studies would be meaningless. Supposing the connected function is:
  (1) $$F\left(w_{i},w_{j}\right)=\left\{ \begin{array}{cc} 0 & \text{disconnected}\\ 1 & \text{connected} \end{array}\right.$$
(2) Network coverage: A comprehensive network topology must ensure that all nodes are covered. Supposing the coverage function is:
  (2) $$C\left(w_{i},w_{j}\right)=\left\{ \begin{array}{cc} 0 & \text{discovered}\\ 1 & \text{covered} \end{array}\right.$$
(3) Residual energy: The topology of a wireless sensor network can be regulated by considering the residual energy of the nodes. When nodes communicate with others, it is likely to become hampered if the nodes display varying residual energy levels, leading to energy depletion and eventually resulting in a failure to communicate. Thus, the node with higher energy reserves ought to be utilized for forwarding. To reflect the residual energy balance of nodes more realistically $\bar{E_{i}\left(w_{i}\right)}$ is used to represent the average value of residual energy of neighbor node j of node i, and nodes with more residual energy serve as neighbor nodes to forward data.
  (3) $$\bar{E_{i}\left(w_{i}\right)}=\frac{1}{m}\sum_{j=1}^{m}\frac{E_{r}\left(j\right)}{E_{o}\left(j\right)}$$
  Where is the residual energy of node *j*, $E_{o}\left(j\right)$ is the initial energy of node *j*.
(4) Energy consumption: Due to the limited energy of nodes used for communication in wireless sensor networks, the energy consumption model is utilized from the literature [18] to avoid network disconnection caused by excessive energy consumption. The energy consumption $E_{s}\left(l,r\right)$ required by a node to send l packets of data is:
  (4) $$E_{s}\left(l,r\right)=E_{elec}\times l+e^{\alpha (f)\cdot \ln10\times r}\times C\times r\times T$$
  (5) $$a\left(f\right)=0.11\frac{f^{2}}{1+f}+44\frac{f^{2}}{4100+f^{2}}+2.75\frac{f^{2}}{10^{4}}+3\times 10^{-3}$$
  (6) $$C=2\pi \times \frac{p^{2}}{\rho \cdot c_{equivalent}}\times 10^{-9.5}$$
  Where *r* is the communication radius of the node, *l* is the size of the data packet, $E_{elec}$ is the energy consumed to receive 1 bit of data, *T* is the time required for data transmission, *a*(*f*) is the medium absorption factor, p is the pressure, ρ is the density of water and is the equivalent sound speed.
  The energy consumption $E_{r}\left(l\right)$ required by the node to receive a data packet is:
  (7) $$E_{r}\left(l\right)=E_{elec}\times l$$
(5) The signal-to-interference-plus-noise ratio (SINR) plays a pivotal role in measuring the quality of data transmission in wireless sensor networks as shown in Eq. (8):
  (8) $$\gamma_{ij}=\frac{2B_{n}}{R_{ij}}.p_{ij}.\frac{A^{-1}\left(r_{ij},f\right)}{\sigma^{2}+\sum_{k=1}^{m}\mu_{ik}p_{ik}r_{ik}^{-a}10^{-\frac{\alpha \left(f\right)r_{ik}}{1000}\times 0.1}}$$
  (9) $$A^{-1}\left(r_{ij},f\right)=r_{ij}^{-a}.10^{-\frac{\alpha \left(f\right)r_{ij}}{1000}\times 0.1}$$
  Where this is the rate of signal transmission, this is the bandwidth of the network, the as node *I* into a total of *m* groups, this the noise variance, this the communication power, the $r_{ij}$ is the distance of signal transmission, α(f) is the medium absorption factor, the *f* is the frequency at which the signal is transmitted.
(6) Data transmission success rate: When the data packets in the wireless sensor network are large, the transmission success rate is shown in formula (10):
  (10) $$Suc\left(i,j\right)=f\left(\gamma_{ij}\right)\approx {\left(1-\frac{e^{-\frac{{}_{\gamma_{{}_{ij}}}}{2}}}{\sqrt{2\pi_{\gamma_{{}_{ij}}}}}\right)}^{l}$$
  Where $\gamma_{ij}$ the signal-to-interference plus noise ratio, *l* is the size of the data packet.

## Framework design

### Ordinal potential game model

By considering sensor nodes as game participants and their available power sets as the strategy set, a wireless sensor network can be regarded as a strategy game. The ordinal potential game is a type of strategy game that comprises three elements: game participants, strategy set, and payoff function. The detailed meanings of each element are as follows:

(1) Game participants *N*: N={1,2,···,n} is the wireless sensor node, and *n* is the number of participants in the game.
(2) Strategy set *W*: $W=W_{1}\times W_{2}\times \cdot \cdot \cdot \times W_{n}$ is the Cartesian product of strategy set, Where is the set of optional strategies for participant *i*, and denotes the *k* strategies available for participant *i*. A particular strategy choice is generally denoted by, Where denotes the strategy of *i* and denotes the strategies of participants other than participant i.
(3) Revenue function *U* (*w*): $U=\{u_{1},u_{2},\cdot \cdot \cdot ,u_{n}\}$ denotes the return function, which $U_{i}\left(w_{i},w_{-i}\right):W\to R$ denotes the return of participant i under the strategy$\left(w_{i},w_{-i}\right)$.

Definition 1 Nash equilibrium: If the game modelG={N,W,U(w)}, for$\forall i\in N,\forall w_{i}\in W{}_{i}$, there are:

(11) $$U_{i}\left(w_{i}^{*},w_{-i}^{*}\right)\geq U_{i}\left(w_{i},w_{-i}^{*}\right)$$

Where $w_{i}^{*}$ is the optimal strategy of participant *i* to other participants, and$w_{-i}^{*}$ is the optimal strategy of participants other than *i*? Then is a Nash equilibrium of the gameG={N,W,U(w)}.

Definition 2 ordinal potential function: For the strategy gameG={N,W,U(w)}, when$\forall i\in N,\forall w_{-i}\in W{}_{-i},w_{i}^{1},w_{i}^{2}\in W_{i}$, the function, there are:

(12) $$\text{sgn}\left(U_{i}\left(w_{i}^{1},w_{-i}\right)-U_{i}\left(w_{i}^{2},w_{-i}\right)\right)\Leftrightarrow \text{sgn}\left(\Lambda \left(w_{i}^{1},w_{-i}\right)-\Lambda \left(w_{i}^{2},w_{-i}\right)\right)$$

Then the function is the ordinal potential function of theG={N,W,U(w)}.

Theorem 1 *If*G={N,W,U(w)}*is an ordinal potential game, the function is an ordinal potential function, and if there is*w∈W*that makes it optimal, then w is a Nash equilibrium of G*.

Definition 3 Pareto optimal: If no strategy can obtain Eq. (13), at least one participant satisfies Eq. (14), then the strategy is praetor optimal.

(13) $$U_{i}\left(w_{i}^{*},w_{-i}^{*}\right)\leq U_{i}\left(w_{i},w_{-i}^{*}\right)$$

(14) $$U_{j}\left(w_{j}^{*},w_{-j}^{*}\right)\leq U_{j}\left(w_{j},w_{-j}^{*}\right)$$

Definition 4 Node degree: If the set of neighboring nodes in the wireless sensor network that can communicate with sensor node *i* is *B*, then the node degree *K* is the number of nodes in the set, namely *K*=|*B*|. The average node degree is the ratio of the node degree *K* to the total number of sensor nodes n in the network, namely |*B*|/*n*.

### Theil entropy measure

Theil entropy measure is a metric in economics that employs entropy to measure the inequality level among individuals or groups. The aforementioned concept is represented by the expression in Eq. (15):

(15) $$T=\sum_{i=1}^{n}\frac{y_{i}}{\sum_{j=1}^{n}y_{j}}\cdot \ln\frac{y_{i}}{\bar{y}}$$

Where the income of *i*th individual is the average of the total income of the *n* individuals.

The concept of entropy frequently serves as a measure of the degree of energy equilibrium. To achieve a more precise representation of the residual energy balance and attain energy equilibrium, we introduce Theil entropy measure *T* into the revenue function. The population share is then understood as the residual energy balance, wherein the residual energy of all nodes in T is substituted with the residual energy of neighbor node *j of* node *i* for an accurate output as shown in Eq. (16).

(16) $$T=\sum_{i=1}^{n}\frac{E_{r}\left(i\right)}{\sum_{j=1}^{n}E_{r}\left(j\right)}\cdot \ln\frac{E_{r}\left(i\right)}{\bar{E_{r}}}$$

Where $E_{r}\left(i\right)$ is the residual energy of node *i*,$E_{o}\left(i\right)$ is the initial energy of node *i*, and represents the average of the residual energy. The network load is balanced by adding an equation $\frac{1}{T+1}$ to the gain function. As the game is executed, the value of $E_{r}\left(i\right)$ gradually decreases, the larger the value of the smaller the value of T and the larger the gain function. Therefore, node *i* will prefer neighbor node *j* with more remaining energy to perform the forwarding task.

(1) Improved Return Function Based on Theil Entropy Measure

Due to the harsh environment of random deployment of wireless sensor nodes, it's not easy to design realistic models. However, the key part of the model design is the establishment of the gain function; this paper combines the elements mentioned before to design the gain function in line with the game modelG={N,W,U(w)}, which is described as follows:

(17) $$\left\{ \begin{array}{c} U_{1i}\left(w_{i},w_{-i}\right)=F_{i}\left(w_{i},w_{-i}\right)C_{i}\left(w_{i},w_{-i}\right)\cdot [\alpha E_{i}^{t\max}\frac{1}{T+1}+\beta \bar{E_{i}\left(w_{i}\right)}+\lambda Suc_{i}\left(w_{i},w_{-i}\right),E_{r}\left(i\right)\geq 0.2E_{o}\left(i\right)\\ U_{2i}\left(w_{i},w_{-i}\right)=F_{i}\left(w_{i},w_{-i}\right)C_{i}\left(w_{i},w_{-i}\right)-\frac{E_{i}^{t}}{E_{i}^{t\max}},E_{r}\left(i\right)<0.2E_{o}\left(i\right) \end{array}\right.$$

Where $U_{1i}\left(w_{i},w_{-i}\right)$ is the primary gain function,$U_{2i}\left(w_{i},w_{-i}\right)$ is the secondary gain function, α,β,λ is the weight factor and all are positive, $F_{i}\left(w_{i},w_{-i}\right),C_{i}\left(w_{i},w_{-i}\right)$ is the connectivity and coverage of the network, $E_{i}^{t\max}$ is the maximum energy consumption of node i, T is the Thiel entropy criterion, $\bar{E_{i}\left(w_{i}\right)}$ is the mean value of the residual energy of the node over the initial energy of the upper node, $Suc_{i}\left(w_{i},w_{-i}\right)$ is the data transmission success rate, $E_{i}^{t}$ is the energy consumption of the node at the current policy.

As the game is executed, the residual energy of a node decreases. We use the Theil entropy measure *T* to express the equilibrium of the nodes' residual energy in this paper. If the residual energy of neighboring nodes of node *i* is more, the smaller the value of *T* and the larger the gain function. To reduce the energy consumption during the game and prolong the life cycle of the network, when the remaining energy of node *i* is$E_{r}\left(i\right)>0.2E_{o}\left(i\right)$, the profit function$U_{1i}\left(w_{i},w_{-i}\right)$ that comprehensively considers multiple factors is adopted. On the contrary, it adopts$U_{2i}\left(w_{i},w_{-i}\right)$, which takes the connectivity and coverage of nodes as the key income function for evaluating income.

(2) Improved Model Proof

The improved game model G={N,W,U(w)} is the ordinal potential game, and its function Λ:W→R is the ordinal potential function:

(18) $$\Lambda \left(w_{i},w_{-i}\right)=\sum_{i\in N}\left(F_{i}\left(w_{i},w_{-i}\right)C_{i}\left(w_{i},w_{-i}\right)\cdot \alpha E_{i}^{t\max}\frac{1}{T+1}+\beta \bar{E_{i}\left(w_{i}\right)}+\lambda Suc_{i}\left(w_{i},w_{-i}\right)-\alpha E_{i}^{t}De_{i}\left(w_{i},w_{-i}\right)\frac{1}{T+1}\right)$$

Proof According to Eq. (17), supposing the difference of income function corresponding to node *I* under different strategies $w_{i}^{1}$ and $w_{i}^{2}$ are shown in Eqs. (19) and (20):

(19) $$\begin{array}{ccc} \Delta U_{i} & = & U_{i}\left(w_{i}^{1},w_{-i}\right)-U_{i}\left(w_{i}^{2},w_{-i}\right)\\ & = & F_{i}\left(w_{i}^{1},w_{-i}\right)C_{i}\left(w_{i}^{1},w_{-i}\right)\cdot \left[\alpha E_{i}^{t\max}\frac{1}{T+1}+\beta \bar{E_{i}\left(w_{i}^{1}\right)}+\lambda Suc_{i}\left(w_{i}^{1},w_{-i}\right)\right]-\alpha E_{i}^{t}De_{i}\left(w_{i}^{1},w_{-i}\right)\frac{1}{T+1}\\ & & -F_{i}\left(w_{i}^{2},w_{-i}\right)C_{i}\left(w_{i}^{2},w_{-i}\right)\cdot \left[\alpha E_{i}^{t\max}\frac{1}{T+1}+\beta \bar{E_{i}\left(w_{i}^{2}\right)}+\lambda Suc_{i}\left(w_{i}^{2},w_{-i}\right)\right]-\alpha E_{i}^{t}De_{i}\left(w_{i}^{2},w_{-i}\right)\frac{1}{T+1}\\ & = & \alpha E_{i}^{t\max}\frac{1}{T+1}\left[F_{i}\left(w_{i}^{1},w_{-i}\right)C_{i}\left(w_{i}^{1},w_{-i}\right)-F_{i}\left(w_{i}^{2},w_{-i}\right)C_{i}\left(w_{i}^{2},w_{-i}\right)\right]\\ & & +\beta \left[F_{i}\left(w_{i}^{1},w_{-i}\right)C_{i}\left(w_{i}^{1},w_{-i}\right)\bar{E_{i}\left(w_{i}^{1}\right)}-F_{i}\left(w_{i}^{2},w_{-i}\right)C_{i}\left(w_{i}^{2},w_{-i}\right)\bar{E_{i}\left(w_{i}^{2}\right)}\right]\\ & & +\lambda \left[F_{i}\left(w_{i}^{1},w_{-i}\right)C_{i}\left(w_{i}^{1},w_{-i}\right)Suc_{i}\left(w_{i}^{1},w_{-i}\right)-F_{i}\left(w_{i}^{2},w_{-i}\right)C_{i}\left(w_{i}^{2},w_{-i}\right)Suc_{i}\left(w_{i}^{2},w_{-i}\right)\right]\\ & & -\alpha E_{i}^{t}\frac{1}{T+1}\left[De_{i}\left(w_{i}^{1},w_{-i}\right)-De_{i}\left(w_{i}^{2},w_{-i}\right)\right] \end{array}$$

(20) $$\begin{array}{ccc} \Lambda & = & \Lambda \left(w_{i}^{1},w_{-i}\right)-\Lambda \left(w_{i}^{2},w_{-i}\right)\\ & = & \sum_{i\in N}\left\{F_{i}\left(w_{i}^{1},w_{-i}\right)C_{i}\left(w_{i}^{1},w_{-i}\right)\cdot \left[\alpha E_{i}^{t\max}\frac{1}{T+1}+\beta \bar{E_{i}\left(w_{i}^{1}\right)}+\lambda Suc_{i}\left(w_{i}^{1},w_{-i}\right)\right]-\alpha E_{i}^{t}De_{i}\left(w_{i}^{1},w_{-i}\right)\frac{1}{T+1}\right\}\\ & & -\sum_{i\in N}\left\{F_{i}\left(w_{i}^{2},w_{-i}\right)C_{i}\left(w_{i}^{2},w_{-i}\right)\cdot \left[\alpha E_{i}^{t\max}\frac{1}{T+1}+\beta \bar{E_{i}\left(w_{i}^{2}\right)}+\lambda Suc_{i}\left(w_{i}^{2},w_{-i}\right)\right]-\alpha E_{i}^{t}De_{i}\left(w_{i}^{2},w_{-i}\right)\frac{1}{T+1}\right\}\\ & = & \Delta U_{i}+\sum_{j\in N,j\neq i}\left\{\begin{array}{c} \left[F_{j}\left(w_{i}^{1},w_{-i}\right)C_{j}\left(w_{i}^{1},w_{-i}\right)-F_{j}\left(w_{i}^{2},w_{-i}\right)C_{j}\left(w_{i}^{2},w_{-i}\right)\right]\cdot \\ \left[\alpha E_{j}^{t\max}\frac{1}{T+1}+\beta \bar{E_{j}\left(w_{j}\right)}+\lambda Suc_{j}\left(w_{j},w_{-j}\right)\right]-\alpha E_{j}^{t}De_{j}\left(w_{j},w_{-j}\right)\frac{1}{T+1} \end{array}\right\}=\Delta U_{i}+\Delta \Lambda_{-i} \end{array}$$

The $F_{i}\left(w_{i},w_{-i}\right)C_{i}\left(w_{i},w_{-i}\right)$ is monotonically non-decreasing, as shown in Eq. (21):

(21) $$F_{i}\left(w_{i},w_{-i}\right)C_{i}\left(w_{i},w_{-i}\right)\cdot \left[\alpha E_{i}^{t\max}\frac{1}{T+1}+\beta \bar{E_{i}\left(w_{i}\right)}+\lambda Suc_{i}\left(w_{i},w_{-i}\right)\right]\geq \alpha E_{i}^{t}De_{i}\left(w_{i},w_{-i}\right)\frac{1}{T+1}$$

Where $F_{i}\left(w_{i}^{1},w_{-i}\right)C_{i}\left(w_{i}^{1},w_{-i}\right),F_{i}\left(w_{i}^{2},w_{-i}\right)C_{i}\left(w_{i}^{2},w_{-i}\right)$ is monotonically identical to$w_{i}^{1},w_{i}^{2}$, and $\Delta \Lambda_{-i}\geq 0$, gets $\text{sgn}\left(U_{i}\right)=\text{sgn}\left(\Lambda_{i}\right)$, that always has the same sign as $\Lambda (w_{i},w_{-i})$. In conclusion, according to definitions 5 and 6, the ordinal potential game Λ is the ordinal potential function, and there must be Nash equilibrium.

## PG-ORTC algorithm

### Initialization stage

The communication radius *r*(*i*) of each sensor node is initialized to the maximum value and the “Hello” broadcast packet is sent by node *i*. The 'Hello' broadcast packet is composed of node ID number, location, synchronization packet, and remaining energy. After receiving the packet, neighbor node j returns an ACK packet to transmitting node *i*. The ACK packet is composed of node ID number, location, synchronization packet, remaining energy, energy consumption, transmission success rate, and profit value. The key part of this phase is to collect the “Hello” broadcast packets sent and the ACK packets received between the nodes. At the same time, the set of neighboring nodes, link sets, and policy sets of node *i* are determined and the network topology G is established.

### Topological gaming stage

After the topology G is initialized, the set of the remaining energy and optional power is obtained by the current power. Each node selects the node of low power compared with the current node according to the policy and checks whether the revenue obtained increases. If this node is updated as to the current node the comparison continues. Otherwise, this node is left unchanged. In addition, there is only one node for adjustment in each round. When the power of each node is the optimal power, there is no change of any node to make the benefit of the whole sensor network greater, and then the network reaches a Nash equilibrium.

### Topology maintenance stage

As the game progresses, each node has different amounts of data to forward and consumes varying levels of energy because of the complex and unpredictable wireless environment, resulting in the network frequently depleting its energy and becoming disconnected. The network topology maintains robustness to adjust itself and maintain normal operation. However, the excessive pursuit of network robustness certainly results in noteworthy energy consumption. The proposed algorithm aims to balance the energy consumption of the nodes in the network and extend the network's lifespan. This is achieved by utilizing Theil entropy measure *T* to assess the residual energy consumption and a segmentation function to determine the gain. Designing a thresholdς, when the ratio of node residual energy to initial energy is greater than the threshold ς, that is $\varsigma >E_{r}/E_{0}$, keeping the network topology unchanged. At this time, if the energy is sufficient at the beginning of network operation, consider all factors affecting the network topology and choose the gain function $U_{1i}\left(w_{i},w_{-i}\right)$. If the remaining energy of the node is poor, consider the connectivity and coverage of the node to choose the revenue function $U_{2i}\left(w_{i},w_{-i}\right)$. When reconstructing the topology to reduce the load of the nodes in the network and extend the network life cycle.

### PG-ORTC algorithm

The pseudo-code for the gaming process is shown in Table 1.

**Table 1:** The algorithm pseudo-code.

**STAGE 1: Initialization parameters** 01: node i sends “Hello” broadcast packets 02: Return the collection of neighbor nodes 03: Determine the policy set for node i
**STAGE 2: Topological game stage** 01:$p_{i}=\{p_{1},p_{2},\cdots ,p_{n}$} 02: While ensuring the topology is Nash equilibrium do 03: for i=1,i≤N,i++ do 04: Choose the strategy when the benefits are greatest 05: if $U_{i}^{1}\left(p_{i}^{1},p_{-i}\right)\geq U_{i}\left(p_{i},p_{-i}\right)$ do 06: if $p_{i}$ ensure the topology is Nash equilibrium do 07: update $p_{i}=p_{i}^{1}$ 08: end if 09: end if 10: end for 11: send “Hello” broadcast packets 12: end while
**STAGE 3: Topology maintenance stage** 01: for i=1,i≤N,i++ do 02: if $\varsigma >E_{r}/E_{0}$do 03: if the energy of the node is sufficient do 04: choose $U_{1i}\left(w_{i},w_{-i}\right)$ 05: else do 06: choose $U_{2i}\left(w_{i},w_{-i}\right)$ 07: end if 08: else $\varsigma <E_{r}/E_{0}$ and topological is connected do 09: reselect the power to construct the topology map 10: end if 11:end for

### Complexity analysis of PG-ORTC algorithm

The complexity of an algorithm is a standard measure of its calculation complexity. This section analyses the time complexity of the PG-ORTC algorithm used in this paper, which consists of two stages: the topology game stage and the topology maintenance stage. In the topology game stage, the payoff of each node is calculated using Eq. (18), and their neighbor set is determined based on the payoff. Assuming the maximum number of neighbor nodes of a node is *M*, where 1≤M<n, (*M* being the maximum number of strategies of each node), the algorithm complexity for this stage is denoted as o(M^2^n). The topology maintenance stage utilizes Theil entropy measure *T t*o assess the residual energy consumption. The algorithm complexity for this stage is denoted as o(Mn). The proposed algorithm's complexity is the sum of o(M^2^n) and o(Mn).

### Comparative analysis of simulation experiments

PG-ORTC algorithm is simulated by using the PyCharm Integrated Development Environment to assess its effectiveness in terms of robustness, network link length, average remaining energy of nodes, and life cycle. The algorithm is then compared to 3DK-RNG, DEBA, and EFPC within the same simulation environment. These comparisons were conducted to verify the efficacy of PG-ORTC.

### Simulation parameters

The parameters of the simulation environment are shown in Table 2.

**Table 2:** Simulation parameter settings.

Experimental parameters	Parameter description	Numerical values
Monitoring area ($m^{3}$)	*V*	400×400×400
Packet length (bit)	*L*	2000
Signal propagation rate(Hz)	*f*	700
Wireless acoustic transmission speed (m/s)	*v*	1500
Number of nodes	*N*	80
Initial energy of nodes (J)	$E_{0}\left(i\right)$	300
Residual energy of nodes (J)	$E_{r}\left(i\right)$	–
Threshold of transmitting power $p_{i}$ (W)	$p_{i}^{\min}$	$3.5\times 10^{-5}$
The threshold value of energy	ς	0.2
Signal transmission rate between nodes (bit/s)	$R_{ij}$	2000

### Impact of weighting factors on performance

Before the comparative analysis, the weight factor α,β,λ of the PG-ORTC algorithm should be determined. In this paper, eighty sensor nodes are subsequently deployed in the monitoring area to restrict any two of the three factors to determine the effect of the other factor on the performance of the algorithm.

**Fig. 1:**
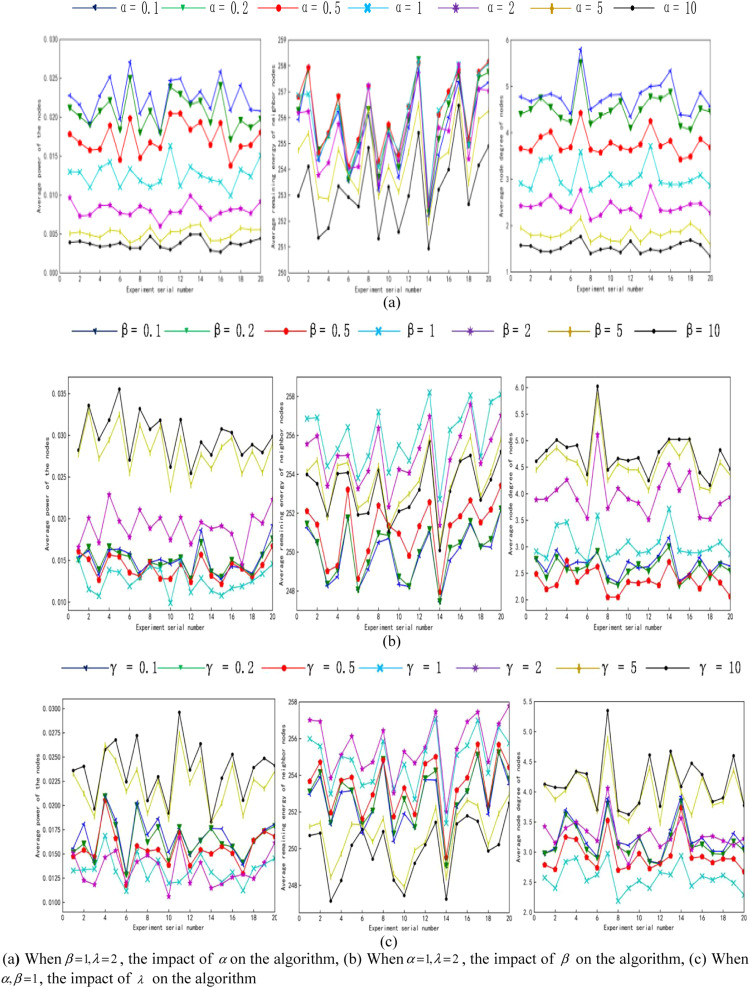
Impact of weighting factors on the algorithm

It can be seen that the average power of nodes decreases with increasing increases with the increasing ofβ,λ, and the change starts to become smaller afterα=1. The residual energy and average node degree of neighboring nodes similarly decrease with the increase ofα, increase with the increase ofβ,λ, and begin to stabilize after in Fig. 1. Therefore, there areα=1,β=1,λ=2, when the node degree is moderate, the transmit power is small, and the topology is more perfect.

### Comparison of robustness

**Fig. 2:**
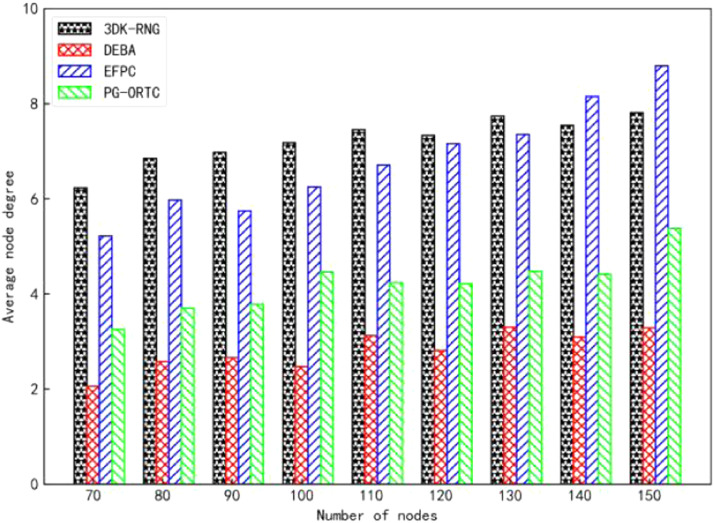
Comparison of average degree of nodes.

**Fig. 3:**
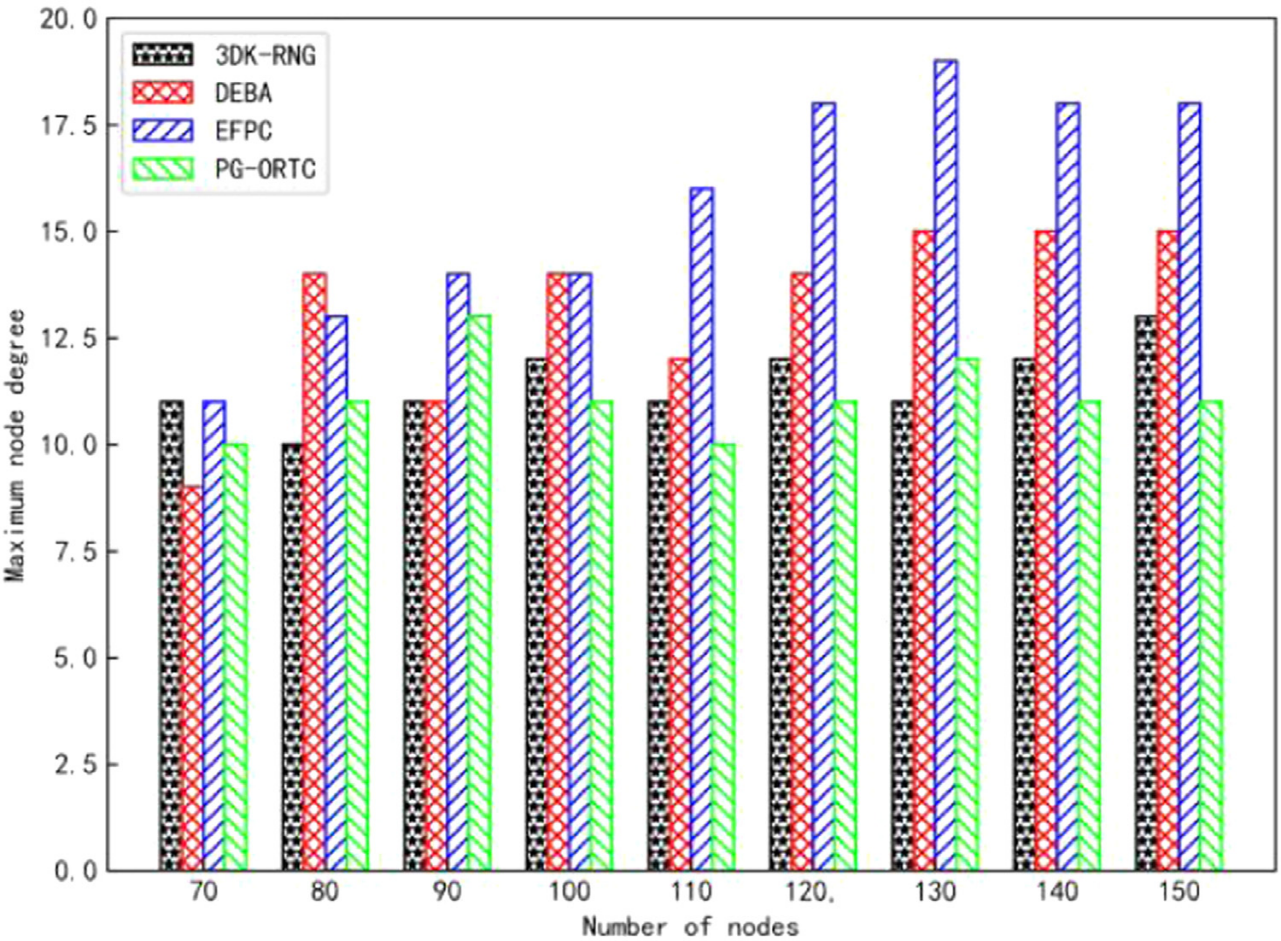
Comparison of maximum node degree.

The sensor nodes were randomly deployed in a monitoring area of 400m×400m×400m, and the robustness of the algorithms was compared by analyzing the topology diagrams of the 3DK-RNG, DEBA, EFPC, and PG-ORTC algorithms, which show the experimental graphs comparing the node degrees of the four algorithms in Figs. 2 and 3. It can be concluded from the figure that the more nodes the greater the degree of nodes. Among them, the average node degree of 3DK-RNG and EFPC are 7.3 and 6.7, and the maximum node degree is 11.3 and 15.5 respectively. However, the average node degree of the PG-ORTC algorithm is 4.3 and the maximum node degree is 9.8, which are both lower than the first two algorithms and higher than the DEBA algorithm. What's more, the node degree is moderate. It does not cause interference in the transmission of data between sensor nodes due to high node degree, requiring multiple retransmissions of data packets and resulting in high-energy consumption. Nor does it result in long communication links between sensor nodes due to low node degree. In addition, this ensures network connectivity and robustness while at the same time-saving energy and providing better performance.

### Comparison of energy consumption

**Fig. 4:**
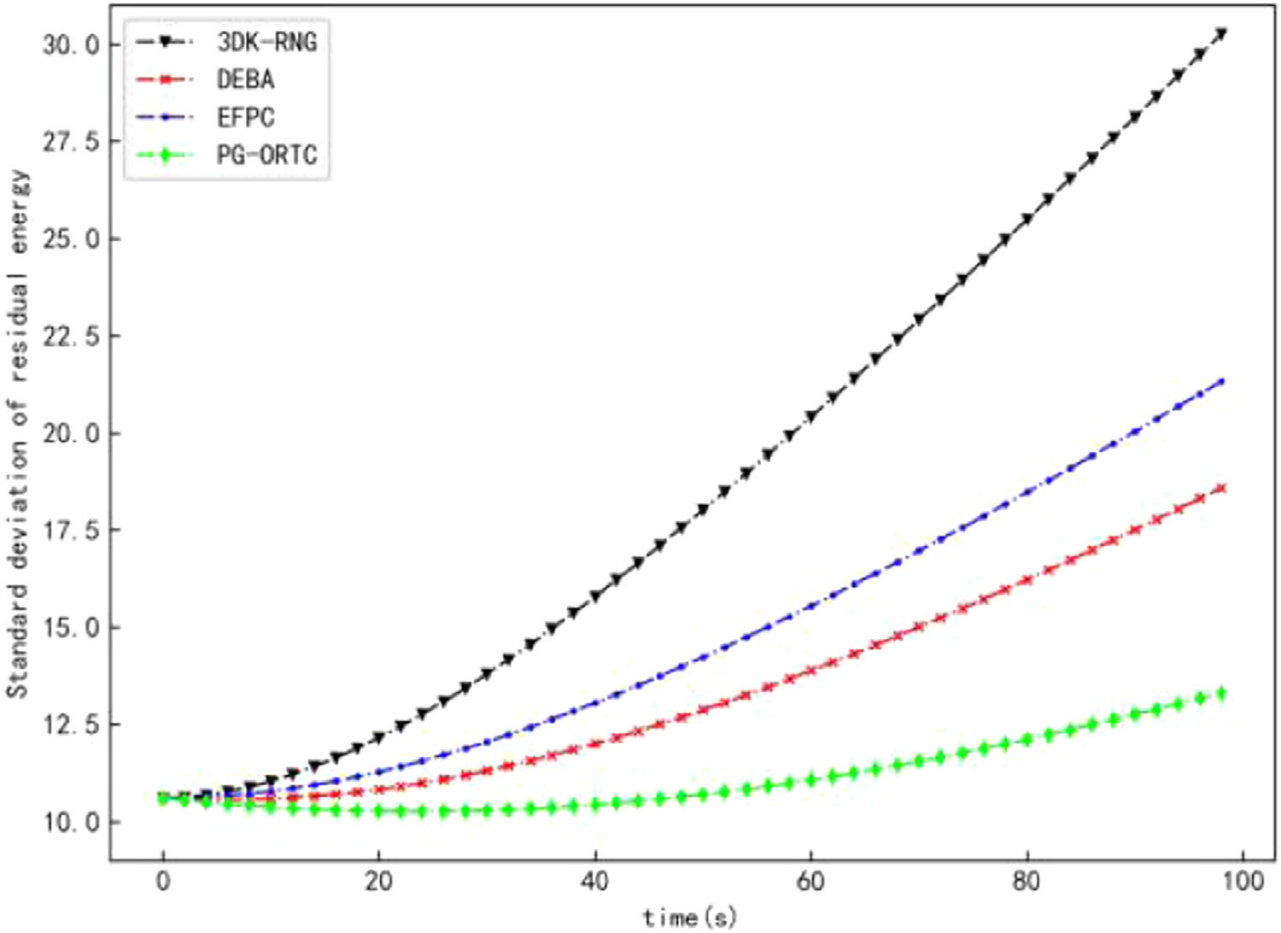
Comparison of standard deviations of residual energy.

As can be seen from Fig. 4, due to the large energy consumption gap between nodes and serious load imbalance, the standard deviation of residual energy of 3DK-RNG and EFPC increases the fastest over time. In addition, the topological network constructed by the DEBA algorithm, although there are relatively fewer redundant links and lower node degrees, there are more isolated nodes, which affects the connectivity of the network. However, the PG-ORTC algorithm effectively balances the energy consumption of the nodes due to the integrated consideration of the nodes' residual energy with that of their neighbors$E_{r}\left(j\right)$, and the introduction of the Theil entropy measure T into the energy consumption function. Besides, the residual energy standard deviation of the nodes grows slowly at the beginning and then grows slowly with time, but it is significantly lower than comparison algorithms.

### Comparison of life cycle

**Fig. 5:**
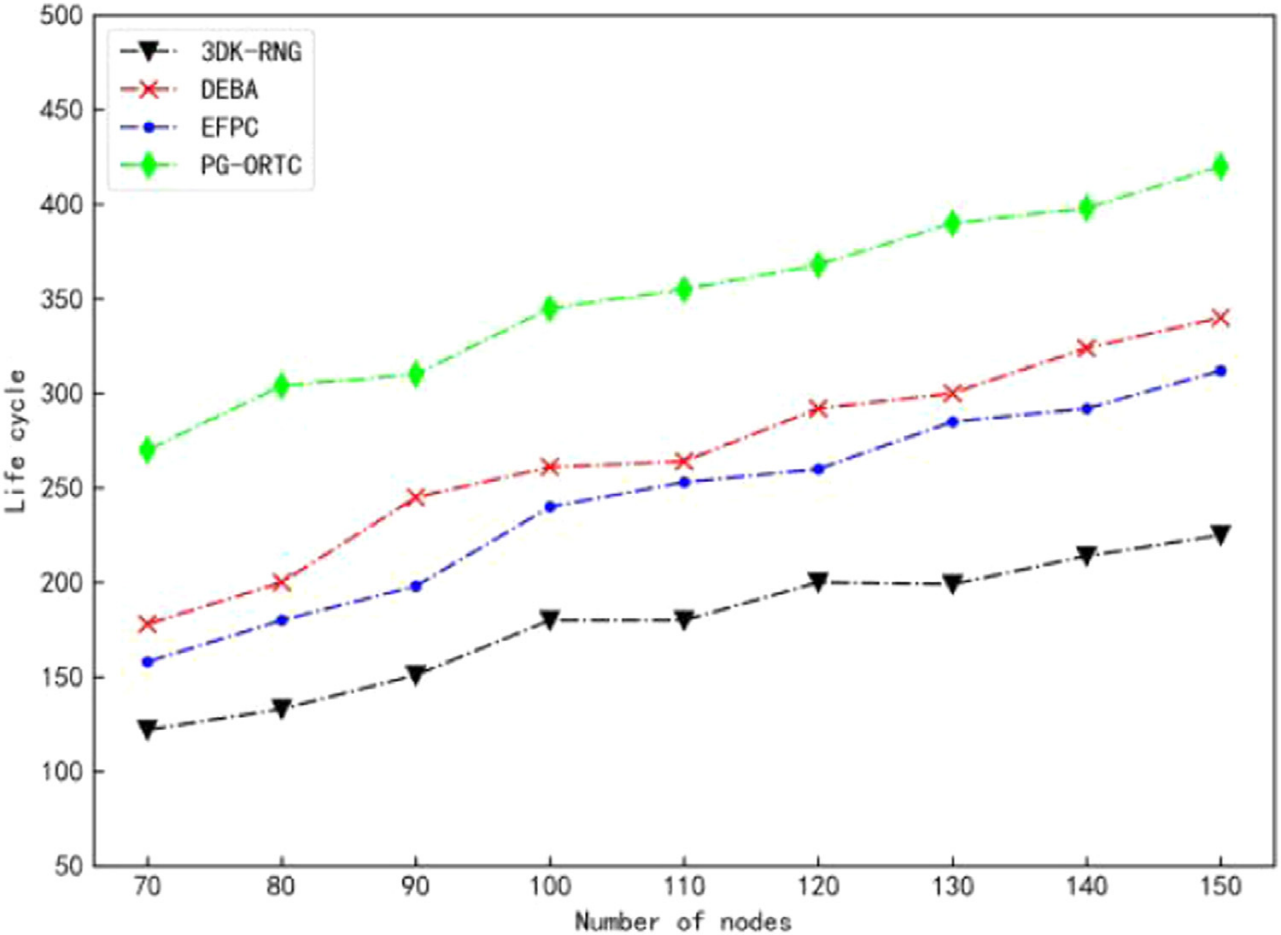
Comparison of life cycles.

**Fig. 6:**
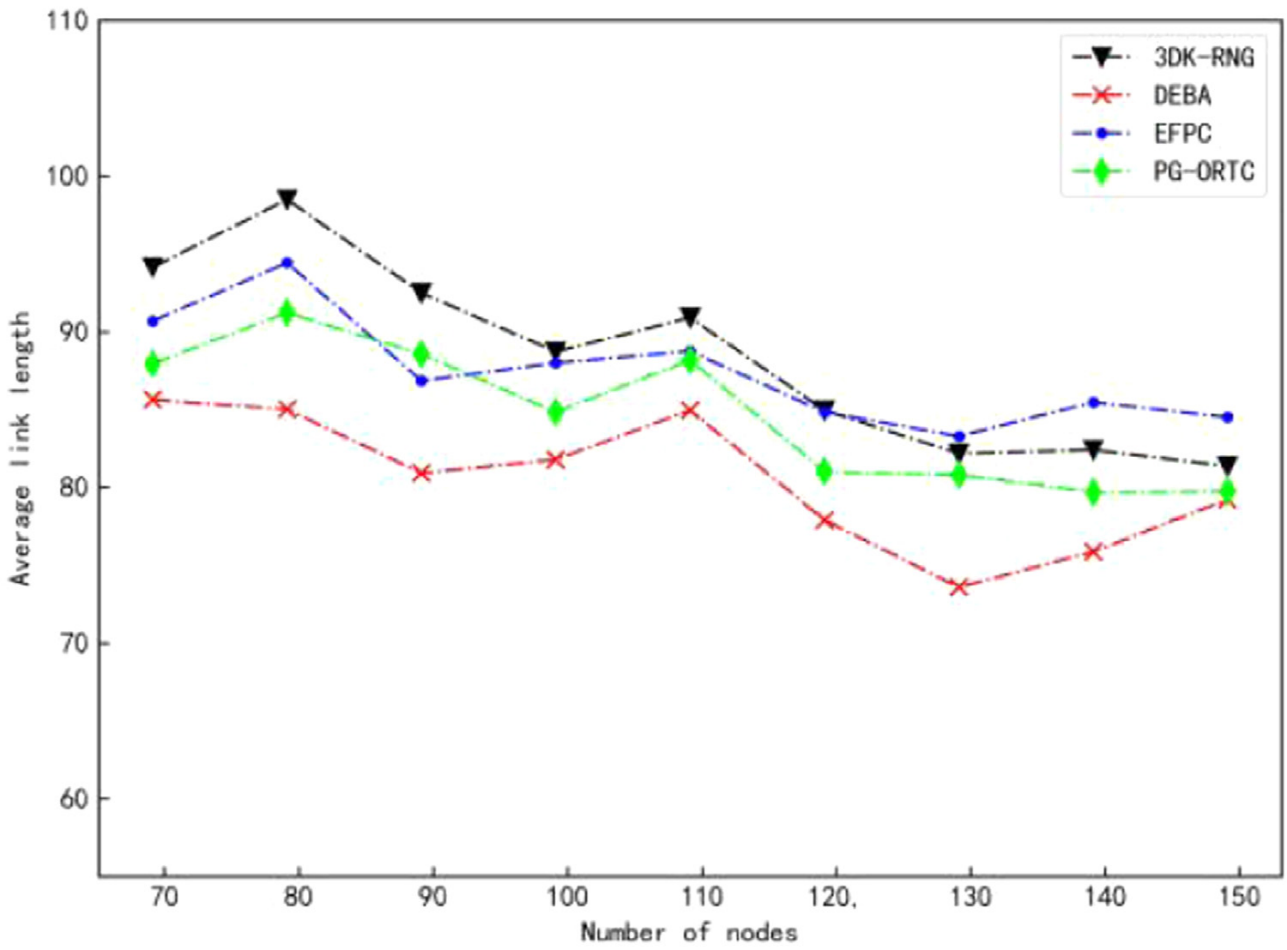
Comparison of average link length.

The comparison of the life cycle changes of 3DK-RNG, DEBA, EFPC, and PG-ORTC algorithms with the increase in the number of nodes is shown in Fig. 5. It can be seen from the figure that the PG-ORTC algorithm has the longest survival period relative to the 3DK-RNG, DEBA, and EFPC algorithms. This is because these three algorithms do not consider the residual energy of neighboring nodes and only consider the robustness of the network. However, the PG-ORT takes into account the residual energy of the node itself and the neighboring nodes and introduces the Theil entropy measure *T* to effectively reduce the node load. Therefore, the lifetime is the longest relative to comparison algorithms (Fig. 6).

### Comparison of link quality

**Fig. 7:**
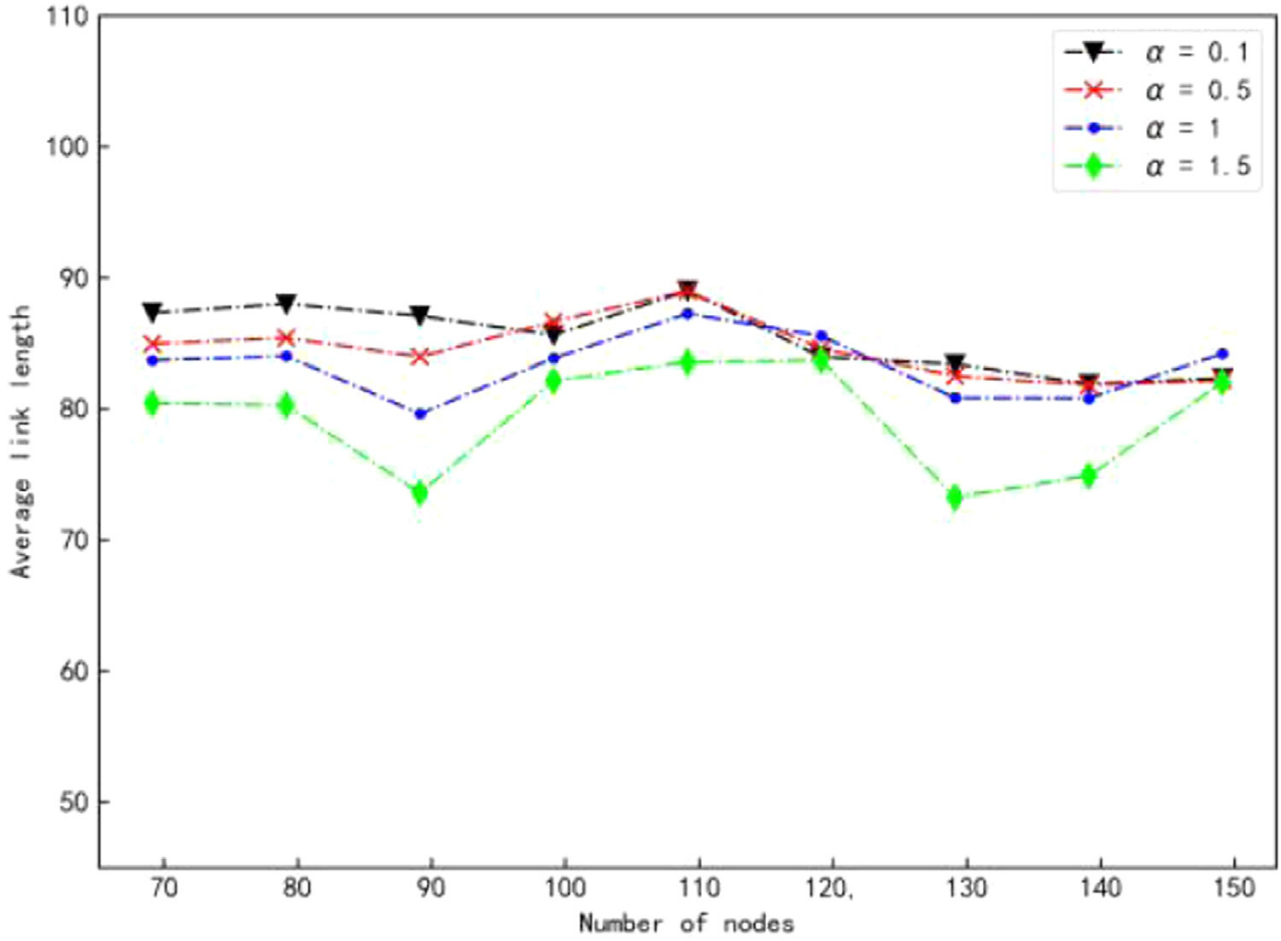
Comparison of link length changes withα.

Changes in link length of 3DK-RNG, DEBA, EFPC, and PG-ORTC algorithms in the same experimental environment are shown in Figure. As can be seen from the figure, the 3DK-RNG algorithm has a higher average link length of the network than the other three algorithms due to more redundant links. What's more, the EFPC algorithm is second only to it. In addition, the DEBA algorithm has the lowest average link length due to the presence of more independent nodes. However, the average link length of the PG-ORTC algorithm proposed in this paper is moderate and the link quality is superior. This shows a comparison of the link lengths of the PG-ORTC algorithm for different numbers of nodes as the number of links changes in Fig. 7. It can be seen that the average link length of the network gradually decreases with the increase of α. With a smaller decrease, the link quality is also relatively improved. Therefore, the network has a certain adaptive degree and can be adjusted to improve the network link quality by adjusting the size ofα.

## Conclusion

This study proposes a solution to the issues related to energy consumption and delay during the transmission process of wireless network signals. The proposed solution integrated the ordinal potential games into wireless sensor network topologies. The Theil entropy measure is used to enhance the revenue function, resulting in an increasing potential function with a unique Nash equilibrium solution that has been theoretically proven. The PG-ORTC algorithm is proposed. The simulation results demonstrate that the algorithm can adjust the weight factor to help the network adapt to different wireless environments and increase the robustness of the network while maintaining wireless sensor network connectivity. This paper comprehensively evaluates the residual energy equilibrium degree between a wireless node and its neighboring nodes. Furthermore, the revenue function is improved by incorporating the Theil entropy measure to assess the performance of the wireless sensor network at different stages. The proposed algorithm effectively reduces the energy consumption of nodes, balances network load and improves the transmission success rate between nodes, as compared to existing gaming algorithms such as 3DK-RNG, DEBA, and EFPC. It achieves high performance by eliminating the need for nodes with low remaining energy to consider comprehensive factors, reducing the incidence of premature deaths, and prolonging the life cycle of the network. Continuous running of nodes will not only lead to high energy consumption but also interfere with the signal transmission within the network. To address this issue, the forthcoming solution will implement the technique of node sleep scheduling within the network topology to enhance the load balancing across the nodes. The algorithm proposed in this paper utilizes an enumerating method to update the node game strategy. However, this leads to a significant amount of redundant game iterations and prolonged processing time, which subsequently increases the computational load and results in a reduced response capability of the algorithm. Therefore, further optimization of the algorithm is required. We will focus on the problem of selecting algorithmic parameters and utilizing machine-learning algorithms to enable an adaptive parameter-tuning mechanism in the future.

## CRediT authorship contribution statement

**Wei Lian Suo:** Methodology, Validation, Data curation, Writing – original draft. **Huan Shen Hao:** Supervision. **Ma Long Yu:** Writing – review & editing.

## Declaration of Competing Interest

The authors declare that they have no known competing financial interests or personal relationships that could have appeared to influence the work reported in this paper.

## Data Availability

Data will be made available on request.
